# Caffeine Ingestion Reverses the Circadian Rhythm Effects on Neuromuscular Performance in Highly Resistance-Trained Men

**DOI:** 10.1371/journal.pone.0033807

**Published:** 2012-04-04

**Authors:** Ricardo Mora-Rodríguez, Jesús García Pallarés, Álvaro López-Samanes, Juan Fernando Ortega, Valentín E. Fernández-Elías

**Affiliations:** Exercise Physiology Laboratory, University of Castilla-La Mancha, Toledo, Spain; University of Sao Paulo, Brazil

## Abstract

**Purpose:**

To investigate whether caffeine ingestion counteracts the morning reduction in neuromuscular performance associated with the circadian rhythm pattern.

**Methods:**

Twelve highly resistance-trained men underwent a battery of neuromuscular tests under three different conditions; *i)* morning (10:00 a.m.) with caffeine ingestion (i.e., 3 mg kg^−1^; AM_CAFF_ trial); *ii)* morning (10:00 a.m.) with placebo ingestion (AM_PLAC_ trial); and *iii)* afternoon (18:00 p.m.) with placebo ingestion (PM_PLAC_ trial). A randomized, double-blind, crossover, placebo controlled experimental design was used, with all subjects serving as their own controls. The neuromuscular test battery consisted in the measurement of bar displacement velocity during free-weight full-squat (SQ) and bench press (BP) exercises against loads that elicit maximum strength (75% 1RM load) and muscle power adaptations (1 m s^−1^ load). Isometric maximum voluntary contraction (MVC_LEG_) and isometric electrically evoked strength of the right knee (EVOK_LEG_) were measured to identify caffeine's action mechanisms. Steroid hormone levels (serum testosterone, cortisol and growth hormone) were evaluated at the beginning of each trial (PRE). In addition, plasma norepinephrine (NE) and epinephrine were measured PRE and at the end of each trial following a standardized intense (85% 1RM) 6 repetitions bout of SQ (POST).

**Results:**

In the PM_PLAC_ trial, dynamic muscle strength and power output were significantly enhanced compared with AM_PLAC_ treatment (3.0%–7.5%; p≤0.05). During AM_CAFF_ trial, muscle strength and power output increased above AM_PLAC_ levels (4.6%–5.7%; p≤0.05) except for BP velocity with 1 m s^−1^ load (p = 0.06). During AM_CAFF_, EVOK_LEG_ and NE (a surrogate of maximal muscle sympathetic nerve activation) were increased above AM_PLAC_ trial (14.6% and 96.8% respectively; p≤0.05).

**Conclusions:**

These results indicate that caffeine ingestion reverses the morning neuromuscular declines in highly resistance-trained men, raising performance to the levels of the afternoon trial. Our electrical stimulation data, along with the NE values, suggest that caffeine increases neuromuscular performance having a direct effect in the muscle.

## Introduction

Athletes' performance is diminished in the early morning and late night in comparison to the afternoon and evenings [1]–[4]. This time-of-day effect on performance occurs during short-term competition events that rely on muscle strength and power output [1], [5], as well as during long term endurance events [6], [7]. The morning reductions in performance can be observed during simple continuous motor tasks (e.g., pedalling [8] or swimming [9]), as well as during complex motor control tasks that involve integration of information (e.g., tennis serve [10] or handwriting [11]). Even though the relationship between the circadian rhythm pattern and the declines in sport performance is well known, the underlying causes for this diminished motor performance are far from being established. This endogenous clock involves variations in basal body temperature and blood concentration of hormones, which in turn could affect body fluids, urinary metabolites excretion and cardiac response (i.e., basal heart rate and blood pressure) [3], [12], [13]. Alternatively, circadian rhythm may affect performance by altering actin-myosin cross bridging processes [14], phosphagen metabolism and/or muscle buffering capacity [12].

To date, studies have mostly described the relationship between these circadian rhythm factors and motor performance. Only a few studies have actually manipulated some of the factors involved in circadian rhythm (e.g., body temperature) to measure variations in neuromuscular performance [15]. This research group found that passive heating somewhat improved morning muscle strength but still it did not reach the neuromuscular performance level found in the afternoon. Likewise, active warm-up, despite raising the morning aural body temperature and increasing muscle force and power output levels, did not increase motor performance to the levels found in the afternoon [4], [16], [17]. These studies suggest that body temperature is one of the most critical components of the circadian rhythm effects on motor performance.

Caffeine is an ergogenic aid commonly used by elite athletes [18], especially since its exclusion from the World Anti-Doping Agency (WADA) prohibited substances list in 2004. This adenosine receptor inhibitor crosses the blood brain barrier and enhances motor performance by maintaining neuro-excitability in the central nervous system of rats [19]. In humans, caffeine enhances muscular endurance [20] and maintains muscle strength after prolonged exercise [21], probably counteracting central nervous system fatigue. Caffeine not only produces an inhibition of phosphodiesterase actions [22], [23] but at ergogenic doses, it could also increase calcium mobilization from the sarcoplasmic reticulum [23]. Studies in tetraplegic participants show that caffeine ingestion delays fatigue by 6% when their paralyzed limbs are electro-stimulated [24]. In addition, caffeine potentiates contraction force when ingested at physiological doses during low-frequency electro-stimulation that produces fatigue [25]. This suggests that caffeine may also have a direct effect on the neuro-muscular junction or in the contractile apparatus itself, since central command is not a factor during electro-stimulation.

There is good evidence that caffeine can improve sprint performance for highly experienced athletes [26], [27], [28] or physically active men [29]. In resistance-trained athletes, muscle strength measured as one-repetition maximum (1RM) is not commonly affected by caffeine ingestion [30], [31]. However, fatigue during repeated contraction at submaximal loads (70% of 1 RM) seems to be delayed by caffeine ingestion although only in lower body musculature [32]. Testing the neuromuscular effects of caffeine ingestion using the 1RM load may be not totally adequate. During the 1RM execution, the time of force application is relatively long and contraction velocity is normally below 0.4 m s^−1^ [33], [34], far from most velocities found during sport actions. Caffeine may enhance contraction velocity and/or motor unit activation against moderate-low resistances for which 1RM may not be a sensitive test to detect it. To our knowledge, no study has addressed whether acute caffeine ingestion could be an ergogenic aid for neuromuscular performance in upper and lower body musculature when performance is measured using submaximal loads that permit high contraction velocity and thus muscle peak power to be reached.

Due to caffeine effects at different loci (i.e., central nervous system, adenosine receptors, Na^+^/K^+^ ATPase activity, intracellular calcium and/or plasma catecholamines concentration [27]), caffeine is likely an effective ergogenic aid to counteract the time-of-day reductions in motor performance. The objective of this study was to deliver caffeine orally at ergogenic doses and observe if it could counter the muscle strength and mechanical power output reductions observed in the morning. Our hypothesis was that caffeine ingestion will increase morning neuromuscular performance in both upper and lower body muscle groups. We hypothesized that caffeine may fully reinstate the level of muscle strength and mechanical power output to the levels found in the afternoon. Finally we hypothesized that the effects of caffeine would be present without affecting basal temperature or the hormonal anabolic blood milieu.

## Methods

### Subjects

Twelve highly resistance-trained men volunteered to participate in this study (age 19.7±2.8 yr, body mass 74.6±2.3 kg, height 173.9±4.8 cm, body fat 11.6±0.8%, resistance training experience 7.2±2.4 yr). Their one-repetition maximum strength (1RM) normalized per kg of body mass was 1.15±0.08 for the bench press (BP) and 1.46±0.15 for the full-squat (SQ) exercises. The subjects were informed in detail about the experimental procedures and the possible risks and benefits of the project. The study complied with the Declaration of Helsinki, was approved by the Bioethics Commission of the University of Murcia, and written informed consent was obtained from each athlete or from their parents prior to participation. Subjects were informed that they could resign from participation at any time during the study. All subjects were light caffeine consumers (≤60 mg d^−1^ from caffeinated soda or lyophilized coffee in milk).

### Experimental design

A randomized, double-blind, crossover, placebo controlled experimental design was used, with all subjects serving as their own controls. Participants underwent the same battery of neuromuscular and biochemical assessments under three different conditions: *i)* morning (10:00 a.m.) with caffeine ingestion (i.e., 3 mg kg^−1^; AM_CAFF_ trial); *ii)* morning (10:00 a.m.) with placebo ingestion (AM_PLAC_ trial); and *iii)* afternoon (18:00 p.m.) with placebo ingestion (PM_PLAC_ trial). Trials were separated by 24 to 36 hours in between. The experimental trials were designed to evaluate the main effects of the time-of-day (morning vs. afternoon) and caffeine ingestion (0 vs. 3 mg kg^−1^) on neuromuscular performance and hormonal responses. We selected those times of day for testing (i.e., 10:00 in the morning and 18:00 in the afternoon) since they are common training schedules for this group of elite athletes that usually perform two-a-day practices.

In the caffeine ingestion treatment (AM_CAFF_), caffeine (Durvitan, Seid, Spain) was provided in gelatin capsules filled to deliver a dose of 3 mg kg^−1^ body mass. The capsules were ingested 60 min before the testing protocol because it has been repeatedly reported that blood caffeine concentration peaks 30–60 min after ingestion [35], [36]. In trials without caffeine ingestion (AM_PLAC_ and PM_PLAC_), the subjects ingested placebo capsules filled with the same amount of dextrose to avoid identification. The amount of additional energy provided by the dextrose (∼2 kcal) was deemed negligible.

All subjects had previously participated in experiments involving the measurement of most of the neuromuscular assessments performed in this study. Nevertheless, participants underwent three familiarization sessions before the start of the experimental trials to avoid the bias of progressive learning on test reliability (one session in the morning (i.e., 10:00 a.m.) and two in the afternoon (i.e., 18:00 p.m.)). The last familiarization session, performed in the morning (10:00 a.m.) of the third day prior to the beginning of each experiment, included the determination of the following variables for each subject (described later in detail): 1) the individual load (kg) that maximizes the muscle power output in the free-weight SQ and BP exercises; 2) the individual load (kg) corresponding to 75% of 1RM in both exercises, and; 3) the intensity of stimuli (i.e., amperage) for electrical stimulation of the quadriceps that elicited an evoked force of 60% of maximal isometric strength.

### Experimental protocol

The day before and during the three days that the experiment lasted, the subjects stayed in the sports research center where they slept and ate all meals. They consumed a diet of 2,800–3,000 kcal·day^−1^, composed of 55% energy intake from carbohydrates, 25% from fat and 20% from protein, evenly distributed across three meals each day (breakfast at 8:30 a.m., lunch at 13:30 p.m. and dinner at 20:00 p. m.).. Subjects refrained from physical activity other than that required by the experimental trials and withdrew from alcohol, tobacco and any kind of caffeine intake 4 days before testing. The day before the onset of the experiment, height was measured to the nearest 0.5 cm during a maximal inhalation using a wall-mounted stadiometer (Seca 202, Seca Ltd., Hamburg, Germany). In every trial, upon arrival to the testing facility, the subjects' body weights were determined and body water estimated in a fasted state using a 4-contact electrode body composition bio-impedance analyzer (Tanita TBF-300A, Tanita Corp., Tokyo, Japan). Following, tympanic temperature (Thermoscan, Braun, Germany) was measured in triplicate after removal of earwax when needed. Next, subjects lay supine for 15 min after which a 9 mL blood sample was withdrawn from an antecubital vein without stasis. A small portion of the whole blood was used to determine hematocrit by triplicate using no-heparinized capillary tubes (70 µL; Hirschmann Laborgerate; Germany) and a micro-centrifuge (Biocen, Arlesa, Spain). The serum and plasma obtained after centrifugation (i.e., 3000 g) was immediately stored at −70°C. Next, the subjects filled out a questionnaire geared to address whether side effects of caffeine were present [18], [37]. Then, subjects ingested the capsules containing either their individualized caffeine dose (3 mg kg^−1^) or the placebo with 330 mL of a fruit milkshake (168 kcals) and a pastry (456 kcals) that served as a standardized breakfast in the AM trials or as an afternoon snack in the PM trial (total of 624 kcals and 68 g of carbohydrate).

**Figure 1:**
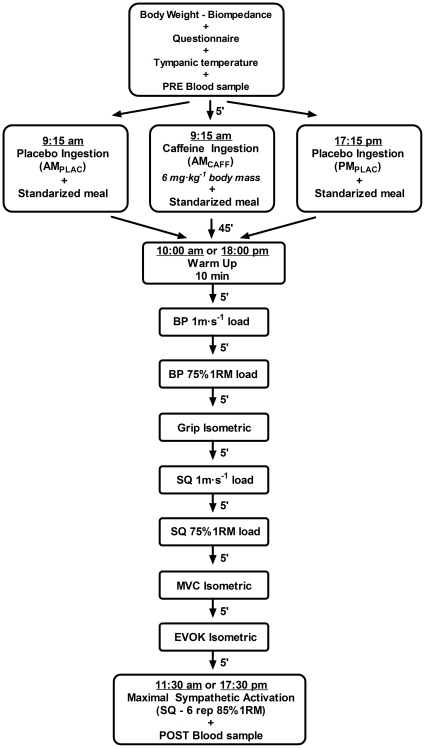
Experimental Protocol. Twelve highly resistance-trained men, in a randomized, double-blind and placebo controlled experimental design, underwent a battery of neuromuscular and biochemical assessments under three different conditions; *i*) morning (10:00a.m.) with caffeine ingestion (i.e., 3 mg kg^−1^; AM_CAFF_ trial); *ii)* morning (10:00a.m.) with placebo ingestion (AM_PLAC_ trial); and *iii)* afternoon (18:00p.m.) with placebo ingestion (PM_PLAC_ trial).

After a standardized warm-up that consisted of 5 min of jogging at 10 km h^−1^ and 5 min of static stretches and joint mobilization exercises, the subjects entered the gym to start the neuromuscular test battery assessments under a strict paced schedule (see Figure 1). These tests consisted of maximum isometric strength as well as the measurement of bar displacement velocity for loads that elicit maximum muscle strength and power output adaptations for upper and lower muscle groups. The battery ended with 1 set of 6 repetitions of full squat exercise with a load of 85% 1RM. This test was designed to elicit a high level of sympathetic stimulation [38]. Each of these tests took the subjects between 8 and 15 seconds to perform. Immediately after this bout, with the subject lying supine, a second antecubital blood sample (9 mL) was rapidly withdrawn. Blood hematocrit and hormone levels (i.e., serum testosterone, cortisol, growth hormone, and plasma nor-and-epinephrine) were evaluated at the beginning of each trial (PRE) and catecholamines at the end of the maximal sympathetic stimulation bout of exercise (POST). Upon completion of the test battery (i.e., ∼90 min from the beginning of the neuromuscular assessments) subjects were discharged and reminded about their schedule for the next trial.

### Maximum dynamic strength and maximal power loads determination

During the last familiarization session the individual loads that elicited a bar displacement of 1.00 m s^−1^ and the load of 75% of 1RM for SQ and BP exercises were identified in a graded loading test using a linear encoder and its associate software (T-Force System, Ergotech, Murcia, Spain, 0.25% accuracy). Loads which elicit a velocity of ∼1.00 m s^−1^ are very close to those that maximize the mechanical power output for isoinertial upper and lower-body multijoint resistance exercises (e.g., free-weight squat or bench press) [33], [39]. In turn, 75% of 1 RM has been described as the minimal load that allows positive adaptations for maximum strength development in highly resistance-trained athletes [40]–[42]. After those loads were individually determined, changes in bar displacement velocity during SQ and BP exercise as a consequence of our treatments (i.e., time-of- day and caffeine ingestion) were measured. Detailed description of the BP and SQ execution technique, as well as the validity and reliability data of the dynamic measurement system (ICC = 1.00; CV = 0.57%) have recently been reported [39] in highly resistance-trained individuals.

### Upper and lower body maximum isometric strength

Maximal isometric voluntary contraction strength on the right knee (MVC_LEG_) was measured as previously described [21] with a validity and intra-day reliability of 0.89 for ICC and 3.4% for CV. Briefly, the subjects sat upright in an adjustable chair with their arms crossed over their chest and fully fastened to prevent extraneous body movements. With the right hip and knee flexed at 90° the right ankle was anchored above the malleolus by a strap connected to a strain gauge dynamometer (Tedea Huntleigh 1263, Germany) interfaced with an A/D board (Powerlab 8SP, ADI) and its related software. Maximal isometric leg strength was collected in triplicate and the best two performances were averaged and recorded for statistical analysis. In turn, subject's arm maximal isometric voluntary contraction strength (MVC_ARM_) was measured in the right hand using a calibrated handgrip dynamometer (Takei 5101, Tokyo, Japan). Participants sat with 0 degrees of shoulder flexion, 90 degrees of elbow flexion and the forearm and hand in a supine position. The best performance out of two repetitions (spaced by 2 min recovery) was recorded for subsequent analysis. The ICC and CV of this measurement were 0.99 and 4.1%, respectively.

### Electrically evoked muscle response

Using the same setting previously described for the MVC_LEG_ testing, the electrically evoked maximal isometric contraction (EVOK_LEG_) of the right knee extensors was measured. Electrical stimulation of the muscle was performed using a four channel high-voltage stimulator (400 V, Megasonic 313, Medicarin, Spain). The stimulus was delivered via three pairs of adhesive patch gel electrodes (4.5×4.5 cm, Medicarin, Spain) placed on the skin above the proximal and distal portions of the vastus lateralis, vastus medialis, and rectus femoris. During preparation for this test, the intensity of stimuli (i.e., amperage; 33–93 mA at each pair of electrodes) was raised progressively until it evoked at least 60% of the participant's MVC [43]. After 4 minutes of rest, we delivered two pairs of trains of 500 ms in duration and 20 Hz in frequency to measure peak evoked force (EVOK_LEG_) at the transducer (expressed in Kg). We chose those electrical stimulation parameters because we have found them to be highly reliable in previous studies from our lab (intra-day CV of 3.5% and intra-day CV of 5.3%; [44]. Because electrodes were removed after each trial, their placing was marked with an antiallergenic permanent marking pen to ensure consistent positioning among trials.

### Maximal sympathetic stimulation bout of exercise

At the end of the test battery in each trial, subjects underwent 1 set of 6 free-weight full squat repetitions at 85% of 1 RM to elicit a high level of sympathetic stimulation [38]. Immediately afterwards, they lay supine and a 9 mL blood sample was rapidly withdrawn. Four milliliters of this blood were mixed in a tube containing 0.4 mL of a solution of reduced glutathione (4.5 mg), sodium heparin (50 IU), and 20 µL of 0.24 M EGTA for determination of plasma epinephrine (E) and norepinephrine (NE) concentration (HPLC with electrochemical detection). Plasma E and NE concentrations were used as an index of whole body sympathetic nerve activation [45].

### Blood concentration of steroid hormones

A portion of the blood was allowed to clot into serum tubes (Z Serum Sep Clot Activator Vacuette®, Greiner Bio-One GmbH, Austria) and then spun at 2000 *g* for 10 min in a refrigerated (4°C) centrifuge (MPW-350R, Med. Instruments, Poland) to separate the serum portion. Serum blood was used to determine total testosterone (TT), cortisol (C) and growth hormone (GH) concentrations using chemiluminescence with an automated analyzer. For TT assessment the blood was processed with an Advia Centaur kit (Bayer Diagnostics, Tarrytown, NY, intra-assay coefficient variation ∼7.7%). For C and GH assessment an immulite 2000 kit was used (Siemens, Los Angeles, Calif., intra-assay coefficients of variation ≤7%).

### Statistical Analysis

Standard statistical methods were used for the calculation of means and standard deviation (SD). Shapiro-Wilk test was used to assess normal distribution of data. Reported sleep quality, fatigue and nervousness perceptions in the questionnaires were not normally distributed and a nonparametric statistical technique was applied. Differences between treatments were analyzed as differences between mean values by using Friedman's two-way rank test. Neuromuscular and biochemical results were analyzed using one-way analysis of variance (ANOVA) for repeated measurements. The Greenhouse-Geisser adjustment for sphericity was calculated. After a significant F test, pairwise differences were identified using Tukey's significance (HSD) post hoc procedure. The significance level was set at p≤0.05. Cohen's formula for effect size (ES) was used, and the results were based on the following criteria; >0.70 large effect; 0.30–0.69 moderate effect; ≤0.30 small effect [46].

## Results

### Pre-testing conditions and caffeine side effects

Before the three battery tests assessments (AM_PLAC_, AM_CAFF_ and PM_PLAC_) body mass and hematocrit were not different although body bio-impedance was lower in the afternoon (18:00 p.m.) compared to both morning trials (p = 0.02; 0.05). The PM_PLAC_ tympanic temperature was significantly elevated when compared to both morning treatments (i.e., 0.7°C; p = 0.02; 0.01; Table 1). Twenty four hours after the conclusion of the AM_CAFF_ trial, the participants did not report problems to sleep or differences in fatigue perception or nervousness in comparison to the AM_PLAC_ or PM_PLAC_ treatments. However, 8.3% of the participants reported gastrointestinal problems and 16.6% an increase in the urinary excretion.

**Table 1:** Physiological conditions before the treatments.

	AM_PLAC_			AM_CAFF_			PM_PLAC_		
Tympanic temperature (°C)	35.3	±	0.6	35.3	±	0.8	36.0	±	0.4* †
Body mass (kg)	74.6	±	2.3	74.8	±	2.2	75.0	±	2.1
Body water (%)	48.2	±	1.3	48.3	±	1.3	48.8	±	1.3
Body fat (%)	11.5	±	0.7	11.7	±	0.9	11.1	±	0.7
Impedance (Ω)	484	±	17	482	±	17	460	±	13* †
Hematocrit (%)	44.8	±	0.7	45.0	±	1.0	44.6	±	0.9

Data are presented as mean ± SD.

* Significant differences compared to the AM_PLAC_ values.

† Significant differences compared to the AM_CAFF_ values. p≤0.05.

### Velocity during loads for maximum dynamic strength and maximal power output adaptations

**Figure 2:**
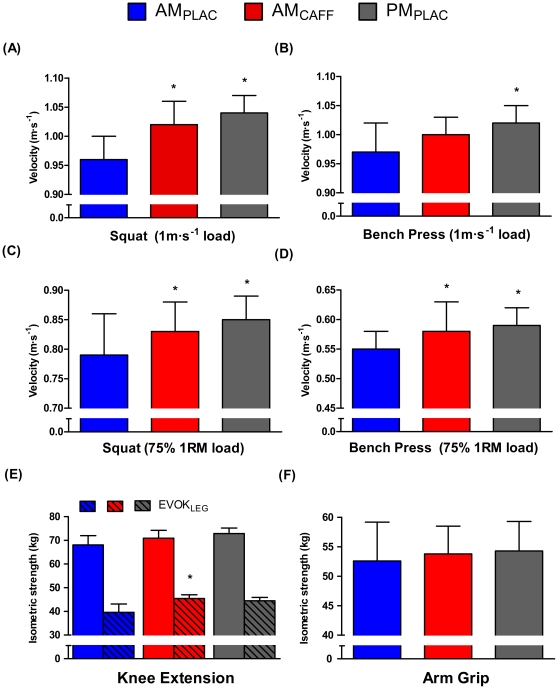
Effects of circadian rhythm pattern and caffeine ingestion on dynamic and isometric maximum strength and muscle power values for upper and lower body actions. A) and B) Velocity for maximal power and; C) and D) Velocity for maximum strength loads for squat and bench press exercises; E) Maximal isometric voluntary contraction strength (MVC_LEG_) and electrically evoked strength (EVOK_LEG_) on the right knee; F) Maximal isometric grip strength. Trials were conducted in the morning (10:00 am) without (AM_PLAC_) or with caffeine ingestion (i.e., 3 mg kg^−1^; AM_CAFF_) and in the afternoon (18:00 pm; PM_PLAC_). Data are means ± SD. *Significant differences compared to the AM_PLAC_ values. p≤0.05.

Velocity for maximal power load (i.e., loads that elicit a bar displacement of 1.00 m s^−1^) in SQ and BP exercises was significantly increased in AM_CAFF_ and PM_PLAC_ treatments when compared to the AM_PLAC_ trial (range of increase of 2.5–7.5%; p = 0.000–0.002; ES from 0.58 to 2.10), except in the BP exercise between AM_CAFF_ and AM_PLAC_ treatments where the difference did not reach statistical significance (p = 0.06; ES = 0.68). Velocity for maximum strength loads (i.e., load of 75% 1RM) in SQ and BP exercises was significantly greater in AM_CAFF_ and PM_PLAC_ than during AM_PLAC_ trial (range of increase of 4.6–6.9%; p = 0.003–0.023; ES: from 0.68 to 1.35; Figure 2C and 2D). However, the velocity during loads for maximal power (1 m s^−1^, Figure 2A and 2B) and maximum strength adaptations (75% 1RM, Figure 2C and 2D) in both exercises were not significantly different between AM_CAFF_ and PM_PLAC_ treatments.

### Maximal isometric voluntary contraction strength and electrically evoked muscle response

No significant differences were detected in maximal isometric voluntary contraction strength of the right knee (MVC_LEG_) among any treatment (AM_PLAC_, AM_CAFF_ and PM_PLAC_). The electrically evoked right leg muscle strength (EVOK_LEG_) was significantly higher following AM_CAFF_ when compared to the AM_PLAC_ trial (16%, p = 0.05, ES = 2.27). Of note, only 7 out of the 12 subjects performed this test due to equipment schedule limitations. No significant differences were observed in the maximum isometric grip strength (MVC_ARM_; Figure 2F) among trials.

### Maximal sympathetic stimulation bout of exercise

**Figure 3:**
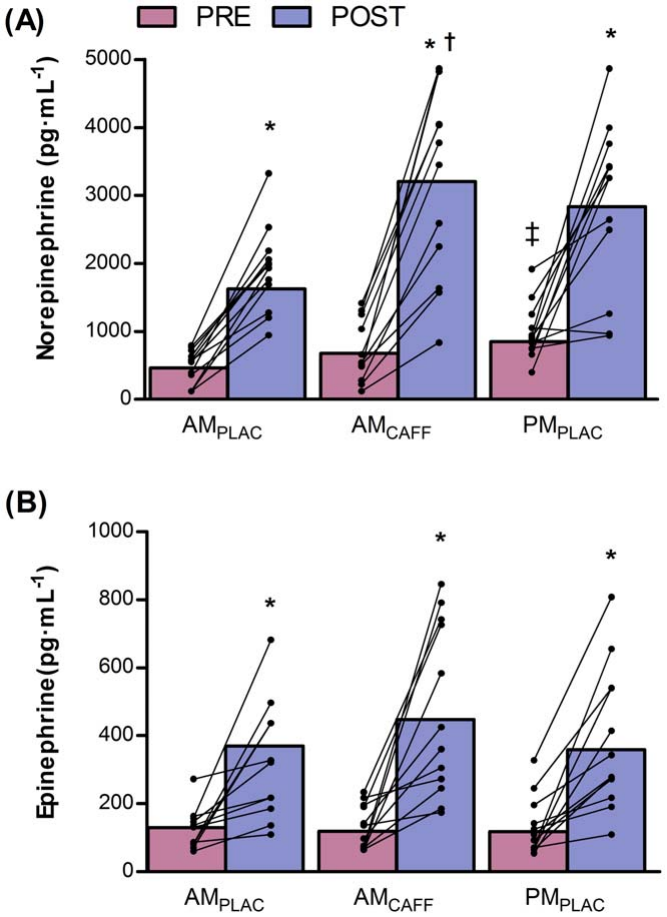
Catecholamine response to a maximal sympathetic stimulation bout of exercise. Norepinephrine and Epinephrine changes following a bout of 6 free-weight squats repetitions with a load of 85% of 1 RM in the morning (10:00 am) without (AM_PLAC_) or with caffeine ingestion (i.e., 3 mg kg^−1^; AM_CAFF_) and in the afternoon (18:00 pm; PM_PLAC_). Data are means ± SD for 12 resistance-trained men. Plasma norepinephrine concentrations reflect whole body sympathetic nerve activation. *Significant differences compared to the PRE values of the same treatment. ‡Significant differences compared to the PRE AM_PLAC_ values. †Significant differences compared to the POST AM_PLAC_ values. p≤0.05.

The increase in plasma catecholamine concentration (i.e., NE and E) induced by 1 bout of 6 free-weight squat repetitions not to failure (85% 1 RM) is shown in Figure 3. Prior to exercise and caffeine ingestion (i.e., PRE) levels of E were similar among trials and increased a similar amount after the bout of intense exercise (2.9, 3.7 and 2.8 fold for AM_PLAC_, AM_CAFF_ and PM_PLAC_, respectively). However, the basal level of NE was higher during the afternoon trial (PM_PLAC_; p = 0.005) than during AM_PLAC_. After the bout of 6 intense squat repetitions (i.e., POST), plasma NE concentration increased 5 fold following the AM_CAFF_ trial which resulted in significant higher levels of NE than in the AM_PLAC_ trial (Figure 3; p = 0.02). This suggests higher whole body sympathetic nerve activity during the AM_CAFF_ trial than in the AM_PLAC_ trial. The final levels of NE following the PM_PLAC_ trial were no different than those reported during the AM_CAFF_ trial, but tended to be higher than those following the AM_PLAC_ treatment (p = 0.07).

### Blood concentration of steroid hormones

Total testosterone, cortisol and growth hormone serum concentrations in the PRE situation (before caffeine ingestion) were not different between the AM_PLAC_ and AM_CAFF_ trials as expected. Testosterone and cortisol levels were lower (p = 0.000–0.006) in the afternoon trial (PM_PLAC_) compared to the AM_PLAC_ and AM_CAFF_ trials (Table 2). In contrast, growth hormone tended to be higher during the PM_PLAC_ than in the morning trials, but the high variability prevented us from finding significant differences. The ratio testosterone-cortisol tended to be higher in the afternoon trial compared to both morning trials (AM_PLAC_ and AM_CAFF_). However, this trend did not reach statistical significance (Table 2).

**Table 2:** Serum blood steroid hormone concentration in the two trials in the morning (9:15 a.m.; AM_PLAC_ and AM_CAFF_) before caffeine was ingested and in the afternoon (17:15 pm; PM_PLAC_).

	AM_PLAC_			AM_CAFF_			PM_PLAC_		
Growth hormone (nmol·L^−1^)	0.49	±	0.53	0.38	±	0.23	1.05	±	1.27
Testosterone (nmol·L^−1^)	16.1	±	5.8	16.1	±	5.0	9.4	±	4.4* †
Cortisol (nmol·L^−1^)	542	±	120	564	±	93	249	±	79* †
T:C ×1000	31.3	±	12.9	29.3	±	9.4	40.6	±	23.4

Data are presented as mean ± SD.

* Significant differences compared to the AM_PLAC_ values.

† Significant differences compared to the AM_CAFF_ values. p≤0.05.

## Discussion

The purpose of this study was to determine the possible interaction between the effects of time-of-day (morning vs. afternoon) and caffeine ingestion on neuromuscular performance (i.e., dynamic and isometric strength as well as muscle power output) in the upper and lower body musculature. Specifically, we sought to investigate if the stimulant actions of caffeine could reverse the reductions in neuromuscular performance observed in the morning. In addition, we set to determine if acute ingestion of caffeine at a dose known to enhance endurance performance (i.e., 3 mg kg^−1^) would also increase muscle power output in highly resistance-trained athletes (i.e., AM_PLAC_ vs. AM_CAFF_ comparison). Our data supports that caffeine is an ergogenic aid for muscle power output in the upper and lower body musculature of resistance-trained individuals. Furthermore, our results suggest that caffeine ingestion in the morning restores neuromuscular performance (muscle strength and power output) of upper and lower muscle groups to levels found in the afternoon trial (i.e., AM_CAFF_ vs. PM_PLAC_ comparison, Figure 2). We consider that these two findings have practical applications during resistance exercise training and performance.

Using our dynamic measurement system attached to a barbell individually loaded to elicit bar displacements of 1 m s^−1^, we found that caffeine ingestion increased the morning SQ and BP muscle power output by 2.5–5.7% (AM_CAFF_ vs. AM_PLAC_; Figure 2A and 2B). Furthermore, morning caffeine ingestion improved the velocity against loads that maximize maximum strength adaptations by 5.3% and 4.6% in SQ and BP respectively (AM_CAFF_ vs. AM_PLAC_; Figure 2C and 2D). Thus, neuromuscular performance (i.e., maximum dynamic strength and muscle power output) improved in a range of 3–6% in the morning after caffeine ingestion, during the most common exercises used for resistance training (i.e., BP and SQ). We think that these strength and power output enhancements induced by 3 mg kg^−1^ of caffeine ingestion (a dose achieved with approximately 2.5 espresso coffees for a 75 kg athlete) have the potential to prevent the morning declines in sport performance, allowing athletes to train and compete at the level of the evening.

A recent meta-analysis defends the existence of an ergogenic effect of caffeine ingestion on maximal voluntary strength but only for knee extensors [16]. In contrast, our data suggest that caffeine ingestion increased similarly upper and lower body morning dynamic maximum strength (Figure 2, albeit from a close to significant finding in BP in panel B). Our subjects were young (∼20 yr), but very experienced resistance-trained individuals (average 7 yrs of training) that used their upper and lower body during training and competition. Importantly, we found a caffeine effect only when the dynamic contraction was used since isometric leg or arm strength were not improved. Out of the 27 studies, included in the Warren and co-workers' meta-analysis, maximum strength was measured using isometric contractions in 21 of them (i.e., 78% of the entries). It is then possible that the lack of ergogenic effect of caffeine in the upper body strength found in the meta-analysis may be due to the large percentage of isometric contraction studies to evaluate the ergogenic effects of caffeine ingestion. Our data contends that acute caffeine ingestion, increases maximal voluntary strength and power output in the upper and lower muscle groups.

Several publications propose that the lower core temperature during the morning in comparison to the afternoon is one of the factors influencing the reduced morning performance. In fact, when body temperature is raised passively by resting in a hot environment [15], or actively by prolonged exercise (>15 min) muscle performance increased to levels near the afternoon trials [6], [16], [17]. Knowing this literature, we attempted to eliminate the effect of core temperature by providing a standardized warm-up lasting 10 min that contained continuous running and calisthenics. Despite the warm-up, a tympanic temperature difference of 0.7° C between morning and afternoon persisted as has been described previously [47]. Of note, the trials AM_PLAC_ and AM_CAFF_ had the same basal tympanic temperature (Table 1) despite significantly different muscle performance (Figure 2). Thus, although part of the differences between the morning and afternoon performance could be due to the differences in core temperature, the effects of caffeine in improving morning muscle performance seems to be predominant in comparison to the effects of core temperature. It is possible that the combination of raising morning core temperature to the afternoon trial levels plus caffeine ingestion could have resulted in larger gains in muscle strength and power. On the other hand, the amount and intensity of exercise required to increase core temperature 0.7°C in our 19°C dry-bulb environment, would have likely been fatiguing and energy depleting. Caffeine ingestion in the morning, a nutritional habit usual for many athletes, probably allows similar enhancement in muscle strength and power output than warming-up through strenuous exercise or passive warming.

Morning reductions in muscle contractility (i.e., the strength divided by the electromyographic activity) suggest that the morning declines in muscle strength are due to peripheral modifications and not to changes in central neural command [15], [48]. We attempt to measure if the effects of caffeine on improving morning performance were also due to a peripheral muscle factor. We electrically stimulated the right leg to contract by delivering the same individualized current intensity in all trials and found a 15% larger increase in force production during AM_CAFF_ vs. the AM_PLAC_ trial (Figure 2E). Our electrical stimulation directly depolarizes the motor units under the skin [44] and thus the increase in isometric force 60 min after 3 mg kg^−1^ of caffeine ingestion is circumscribed to the muscle itself. Although an ergogenic effect of caffeine through maintaining central neural command has been suggested [19], [21] a peripheral effect of caffeine has not been discarded [24], [25]. Our electrical stimulation data suggest that caffeine improves neuromuscular performance by acting directly upon the muscles and is in agreement with the meta-analysis of Warren et al. [20], that found that electrically evoked strength was higher with caffeine ingestion when expressed as percent of maximal voluntary contraction.

After the battery of neuromuscular tests, subjects were required to perform a bout of 6 free-weight squat repetitions at 85% of 1 RM with the aim of markedly raising sympathetic nerve activity. We measure plasma norepinephrine concentration (NE) as our main index of whole body sympathetic activity since it is derived in more than 80% from the spillover of the terminal nerve endings of the motoneurons [45]. An acute bout of resistance exercise has been shown to increase plasma concentration of NE [49]. The magnitude of the increase in NE may be dependent upon the force of muscle contraction, amount of muscle stimulated and volume of resistance exercise [50], [51]. Despite using the same short-bout of resistance exercise in all three trials, we observed that plasma NE rose higher when caffeine was ingested (AM_CAFF_; Figure 3), suggesting a facilitated sympathetic nerve activation. In contrast, plasma epinephrine concentration, which is mostly derived from the adrenal medulla, did not show a larger increase after caffeine ingestion in comparison to the other trials. Although caffeine ingestion has been consistently reported to raise plasma epinephrine during endurance exercise to fatigue [52], a similar increase during a single bout of resistance exercise not to failure is unreported. We did not obtain samples during recovery and thus we cannot discard a delayed increase in plasma epinephrine upon caffeine ingestion. Our electrical stimulation data in conjunction with the plasma NE values suggest that caffeine increases muscle strength and power output through a direct effect in the muscle.

We measured resting serum hormones to have an index of the anabolic-catabolic balance in relation to the performance measured (i.e., muscle strength and power output). Albeit measurements of only resting blood serum hormone concentration have their limitations, their levels have been extensively reported in resistance-training research [38]. Like others [53], [54], we observed higher levels of blood testosterone and cortisol in the morning than in the afternoon (Table 2). However, the ratio testosterone-to-cortisol (T/C) tended to be higher in the afternoon (ES = 0.51) coinciding with the higher levels of muscle strength and power output. In contrast, Teo et al. [5], argue that the T/C ratio does not vary in accordance with the changes across the day in muscle strength and power output. We were aware that a moderately-high carbohydrate diet should be consumed to maintain validity of any observed changes in the ratio of T/C [55]. Our subjects consumed 55% of their daily calories in the form of carbohydrates and in addition we provided a snack prior to every trial which included 68 g of carbohydrate. Additionally, we measure growth hormone and found a trend for increased levels in the afternoon, also suggesting (like with the T/C ratio) a more favorable anabolic state. In conclusion, our blood hormonal data suggest that in the afternoon, despite an absolute decrease in the concentration of free testosterone, the larger decrease in serum cortisol results in a more favorable anabolic state. The contribution of this hormonal milieu to the increased muscle performance observed in the afternoon is currently under investigation.

In summary, the acute ingestion of caffeine (3 mg kg^−1^) reverses the morning reductions in maximum dynamic strength and muscle power output (2.5–7.0%), increasing muscle performance to the levels found in the afternoon (PM_PLAC_ trial). These caffeine ergogenic effects seem to occur only in dynamic and not in isometric muscle contractions, for both the upper and lower body actions, and are independent of body temperature. Our electrical stimulation data, in conjunction with the plasma norepinephrine concentration, suggest that caffeine increases muscle strength and power through an effect directly in the muscle. In conclusion, caffeine ingestion in the morning, an ergogenic aid of common use among athletes, avoids the morning reduction in muscle performance due to circadian rhythm.
